# NK-DC Crosstalk in Immunity to Microbial Infection

**DOI:** 10.1155/2016/6374379

**Published:** 2016-12-20

**Authors:** Rony Thomas, Xi Yang

**Affiliations:** Department of Immunology, Faculty of Medicine, University of Manitoba, 471 Apotex Centre, 750 McDermot Avenue, Winnipeg, MB, Canada R3E 0T5

## Abstract

The interaction between natural killer (NK) cell and dendritic cell (DC), two important cellular components of innate immunity, started to be elucidated in the last years. The crosstalk between NK cells and DC, which leads to NK cell activation, DC maturation, or apoptosis, involves cell-cell contacts and soluble factors. This interaction either in the periphery or in the secondary lymphoid organs acts as a key player linking innate and adaptive immune responses to microbial stimuli. This review focuses on the mechanisms of NK-DC interaction and their relevance in antimicrobial responses. We specifically aim to emphasize the ability of various microbial infections to differently influence NK-DC crosstalk thereby contributing to distinct adaptive immune response.

## 1. Introduction

The implementation of an effective immune response requires recognition of pathogen and consequent induction of innate and adaptive immune systems. Even though adaptive immune system provides a more versatile means of defense by ultimate protection and memory against the pathogen, innate immune system is crucial in the initiation and subsequent direction of adaptive immune responses. NK cells and dendritic cells represent two central components of the innate immune system, both of which play a key role in combating early infection. NK cells provide the first line of defense against a variety of tumors and microbial pathogens. Morphologically they are characterized as large granular bone marrow derived lymphocytes, which represent 10% of peripheral blood lymphocytes. In humans, NK cells are divided, based on their functional and phenotypic properties, into two main subsets, namely, CD56^dim^ and CD56^bright^. CD56^dim^ subset shows enhanced cytotoxic activity and expresses CD16, KIRs (killer cell immunoglobulin-like receptors), and perforin whereas CD56^bright^ subset secretes enormous amounts of cytokines and expresses low levels of perforin and CD16 [1]. Upon stimulation, NK cells secrete large amounts of cytokines and chemokines such as IFN-*γ*, TNF-*α*, GM-CSF, CCL3, CCL4, and CCL5 [2]. NK cells identify their targets through a number of activating and inhibitory receptors on their surface and the balance between these signals controls NK cell activation [3].

DCs are the major antigen-presentation cells (APCs) of the immune system and have a crucial role in both sensing pathogens and tuning the immune responses [4]. They consist of different subtypes and are classified based on their phenotype, location, and function [5]. Conventional DCs (cDCs) mainly reside in the lymphoid tissues such as spleen, thymus, and secondary lymph nodes (LNs). These conventional DCs express higher levels of MHC-II and CD11c and can be further divided into CD8*α*+ and CD8*α*− DCs in mice. When compared with CD8*α*+ DCs which more often induce Th0 cells to elicit Th1 response, CD8*α*− DCs more likely induce Th2 responses [6]. In addition, cDCs in the nonlymphoid tissues such as the intestine and the lung consist of two major subsets: CD103^+^ and CD11b^hi^ DCs. Interestingly, CD103^+^ DC in the nonlymphoid organs including lung, gut, and skin form a unified subset, which is developmentally related to the CD8^+^ cDC in lymphoid organs [7]. This correlation is demonstrated by their shared dependence on certain transcriptional factors such as* Batf3* and* Irf8 *and functional characteristics of antigen cross-presentation. The linkage between CD8^+^ DC and CD103^+^ DC was further strengthened by the reports showing unique common expression of XCR1, a chemokine receptor, by these DC subsets [8, 9]. Since XCR1 are also expressed in human BDCA3^+^ DC and sheep CD26^+^ DC (the equivalents of mouse CD8^+^ DC), the term “XCR1^+^ DC” could be designated to “CD8^+^ type DC” in both lymphoid and peripheral tissues across all mammalian species. Plasmacytoid DCs (pDCs) represent a small subset of DCs that enter the lymph nodes through the blood circulation. Upon activation through Toll-like receptor (TLR)-7 and TLR9 stimulations, pDCs secrete profound amounts of IFN-*α* and several chemokines (CCL3, CCL5, and CXCL10) [10]. In humans, DCs express high levels of MHC II and lack markers such as CD3, CD19/20 and CD56. They can be classified as either myeloid or plasmacytoid [11]. Myeloid DCs (mDCs) correspond to mouse cDCs and express myeloid antigens such as CD11c, CD13, CD33, and CD11b. They are divided into CD1c^+^ and CD141^+^ DCs, which share homology with mouse CD11b^+^ DC and CD8/CD103^+^ DC, respectively. CD14^+^ DCs, originally described as interstitial DCs, are a third subset CD11c^+^ myeloid DC found in tissues and lymph nodes. Human plasmacytoid DCs lack myeloid antigens and express CD123, CD303, and CD304 [11]. DCs reside in an immature form at various portals of pathogen entry. Under steady state conditions, DCs express low levels of MHC and costimulatory molecules. On exposure to pathogens, TLRs and other receptors on surface of DCs recognize molecular patterns associated with microbes, which initiates DC maturation, upregulation of CCR7, and consequent migration to the local draining lymph nodes where interaction with naive T cell occurs. Mature DCs express high levels of MHC and costimulatory molecules which enable them to activate naive T cells in T cell areas of secondary lymphoid organs [12]. Priming and modulation of T cells by DCs involves the interaction of CD80 (B7-1)/CD86 (B7.2) and CD40 with CD28/CTLA4 (CD152) and CD40L on T cells, respectively [13]. In addition, activated DCs produce proinflammatory and immunomodulatory cytokines and chemokines, which shape the pattern of immune responses [14].

## 2. NK-DC Interaction

The bidirectional crosstalk between DCs and NK cells can occur in the periphery or in secondary lymphoid tissues where they interact with each other through cell–cell contact and soluble factors. Interaction of NK cells with DC results in maturation, activation, and cytokine production by both cells.

### 2.1. DCs Induce NK Activation

TLR mediated recognition of pathogen by DC stimulates their maturation and secretion of several cytokines, which can activate NK cells. DC promotes NK cell proliferation, cytokine production, and cytolytic activity mainly through the release of cytokines and cell-cell contacts. In vitro studies have demonstrated a central role for DC-derived IL-12 in the induction of IFN-*γ* producing NK cells. IL-18 produced by DC can further induce the expression of IL-12 receptor on NK cells [15]. IL-15 is another relevant cytokine produced by DC which can stimulate NK cell proliferation, survival, and priming of protective NK cell response [1]. In addition, pDCs secrete profound amounts of type 1 interferon (IFN-*α*/*β*) which induce NK cell cytotoxicity [16]. Furthermore, it is found that upon TLR stimulation, IFN-*β* produced by DC induces IL-15 production by DCs as well as NK cells. This IL-15 can be transpresented by DCs to NK cells as well as cispresented by an NK cell to itself for efficient NK cell activation [17, 18]. It has also been shown that TLR-9 stimulated pDCs promote a selective proliferation of CD56^bright^ NK cell subset [19]. Other soluble factors, such as prostaglandin E2 (PGE2) produced by DC have emerged as a potential regulator of NK-DC crosstalk. It can modulate secretion of the chemokines and cytokines that are involved in NK cell recruitment [20]. NK cell activation by DCs also requires direct cell-to-cell contacts. Even though there are controversial reports regarding formation of stable or transient NK-DC interactions in vivo, it is evident that cell-cell contact is required for the confined secretion of IL-18 at the immunological synapse [21, 22]. In fact, the formation of stimulatory synapses, between DCs and NK cells, promotes DC to secrete preassembled stores of IL-12 towards the NK cell. This synaptic delivery of IL-12 by DCs is required for IFN-*γ* secretion by NK cells [23]. Other interactions that promote NK cell IFN-*γ* production include CXC3CL1 expressed on DCs with its receptor on NK cells and triggering of activation receptors NKp46 and NKG2D [24, 25].

### 2.2. NK Cells Promote DC Activation

It is well known from in vitro studies that activated NK cells release profound amounts of IFN-*γ* and TNF which promote DC maturation [1]. Mature DCs secrete cytokines such as IL-12, IL-6, IL-27, IL-21, IL-23, and TGF-*β* [26]. These cytokine signals provided by NK cell-activated DCs in turn control the adaptive immune responses against infections. On the other hand, activated NK cells also have the ability to kill DCs that fail to undergo proper maturation (“DC editing”) through engagement of the activating receptor NKp30 [27]. NK cells discriminate between mature and immature DCs (iDC) by recognizing low amount of class 1 MHC molecules on the surface of immature DCs. In vitro studies have demonstrated that culture of activated human NK cells with iDCs at low NK/DC ratios promotes DC maturation, whereas a higher NK/DC ratio can result in NK cell-mediated killing of DCs [28].

## 3. NK-DC Crosstalk in Viral Infection

The role of NK-DC crosstalk in controlling viral infection has long been recognized. Studies in human immunodeficiency virus (HIV), hepatitis C virus (HCV) and mouse cytomegalovirus (MCMV) infection provide a strong body of evidence to implicate NK-DC interaction in fine-tuning the adaptive immune response to viral infections.

### 3.1. NK-DC Crosstalk in HIV Infection

Recent evidence suggests that the crosstalk between NK cells and DCs is impaired during HIV-1 infection. In vitro studies by Mavilio et al. have shown that there was a marked impairment in the interactions between CD56^neg^ NK subset and autologous DCs from HIV 1-infected viremic but not aviremic individuals. Defective interaction includes abnormalities in the process of reciprocal NK–DC activation, maturation as well as a defect in the NK cell–mediated editing of immature DCs (iDCs) [29]. This defect in DC editing is mediated by an increase in CD56^neg^ NK cells with impaired NKp30 function. In addition, DCs infected with HIV-1 are resistant to NK-induced TNF-alpha-related-apoptosis-inducing-ligand- (TRAIL-) mediated apoptosis. This resistance occurs due to High-mobility group box 1 (HMGB1) mediated upregulation of two anti-apoptotic molecules, the cellular-Flice like inhibitory protein (c-FLIP) and the cellular inhibitor of apoptosis 2 (c-IAP2) [30]. In fact, in vitro studies suggest that HIV infection induces IL-10 production of DC, which promotes resistance of immature DCs to NK cell-mediated elimination [31]. Moreover, pDCs from HIV-infected individuals are found to have deficiency for producing IFN-*α* and TNF [32]. Similarly, cDCs from HIV-1-infected individuals show impaired secretion of IL-12, IL-15, and IL-18, leading to decreased NK cell activation [33].

### 3.2. NK-DC Crosstalk in HCV Infection

In vitro studies have demonstrated that NK cells from chronic hepatitis C virus infected donors (HCV-NK) express higher levels of NK inhibitory receptor, CD94/NKG2A. On binding of CD94/NKG2A with HLA-E expressed on hepatic cells, HCV-NK cells produce IL-10 and transforming growth factor-*β* (TGF*β*) [34]. IL-10 and TGF*β* are found to act as suppressive factors of DC activation. Studies suggest that IL-10/TGF*β*-modified DCs can induce IL-10-producing T cells as well as CD25^+^CD4^+^ regulatory T cells [35]. The blockade of NKG2A also stimulated NK cells to generate Th1-polarized CD4^+^ T cells. The results suggest that NK cell modulation of DC through NKG2A interferes with the ability of DCs to generate HCV-specific adaptive immune responses. In addition, chronic hepatitis C virus (HCV) infected DC virtually lost the ability of type I IFN-mediated production of IL-15. This defect in IL-15 production leads to reduced expression of MHC class I-related chains A and B (MICA/B) on DCs. MIC-A and MIC-B are ligands for NKG2D, which transduce positive intracellular signals in NK cells to promote NK activation [36]. This explains the reduced frequency of NK cells in individuals with chronic HCV infection [37].

### 3.3. NK-DC Crosstalk in Mouse Cytomegalovirus (MCMV) Infection

During early stage of MCMV infection, pDCs produce IFN-*α*/*β* and IL-12 as a consequence of recognition of viral CpG DNA sequences through the TLR9/MyD88 pathway. The IFN-*α*/*β* and IL-12 cytokines produced by pDCs promote the capacity of NK cells in cytotoxicity, IFN-*γ* production, and functional maturation of cDC for effective antiviral CD8^+^ T cell response [38]. Moreover, CD11b^+^ myeloid DCs also activate NK cells through NKG2D-NKG2D ligand interactions as well as the production of IFN-*α*/*β*, IL-18, and IL-12 [39]. This effect was independent of TLR9 but requires TLR2 and/or TLR3 [40]. In addition, MCMV infected CD8*α*^+^ DCs produce very high levels of IL-12 and IL-18, which specifically expand Ly49H^+^ subset of NK cells [41]. It has been reported that MCMV can induce high IFN-*α*/*β* to promote its own survival by inhibiting cDCs and delaying the response of antiviral effector, CD8^+^ T cells. Interestingly, Ly49H^+^ NK cell−mediated functions during early stage of MCMV infection prevent release of immunosuppressive levels of IFN-*α*/*β* to protect against MCMV-induced loss of splenic CD8*α*^+^ DCs [42]. Altogether, these studies suggest that NK cell and DC interact with each other to create a balance between the positive and negative effects of IFN-*α*/*β* and other cytokines for the optimal control of MCMV infection.

## 4. NK-DC Crosstalk in Parasite Infection

In response to* Plasmodium chabaudi* AS infection in mice, NK cells stimulated DC to mature and produce IL-12 by cell-cell contact and NK cell derived IFN-*γ* [43]. In vitro studies suggest that during infection with* Toxoplasma gondii*, NK-DC interaction leads to elevated IL-12 production by DC which in turn increases their ability to prime CD8^+^ T cell response against the parasite. This effect was due to TLA (Toxoplasma lysate Ag) mediated upregulation of NKG2D receptors such as MULT-1 and RAE-1 on DC. Neutralization of NK activating receptor, NKG2D, in* T. gondii *infected mice led to defect in parasite clearance [44]. Moreover, adoptive transfer studies of preactivated NK cells into* Leishmania amazonensis*-infected mice significantly increased DC and T cell activation as well as reduced tissue parasite loads [45]. Similarly, it is found that, in response to* P. berghei* ANKA infection, NK cells stimulated DC-mediated priming of CD8^+^ T cells. On the other hand, both DC-depletion and genetic deletion of IL-12 in mice almost completely abrogated NK cell-mediated IFN-*γ* responses to* P. berghei* ANKA infection [46]. Moreover, IL-12 has been shown to be essential for the in vitro NK cell-derived IFN-*γ* response to* P. falciparum *[47],* P. chabaudi* [43],* T. gondii* [48], and in vivo NK cell responses to* Leishmania major *[49]. In addition, studies in* P. falciparum *infected RBC [50] and* Leishmania major* infection in mice [51] suggest that NK cell activation also depends on IL-2 production from antigen-specific CD4^+^ T cells apart from DC derived stimuli. These studies highlight the concept that stimulation of adaptive immune response by NK-DC crosstalk enhances NK cell activation and cytotoxicity for optimal immune response.

## 5. NK-DC Crosstalk in Bacterial Infection 

Recent studies suggest that the cooperation between NK cells and DC is also crucial in several antibacterial responses. Initial studies using in vitro systems demonstrated that stimulation of human peripheral NK cells with LPS treated mature DC augmented the cytolytic activity of NK cells. Reciprocally, coculture of NK cells with immature DC induced DC maturation and IL-12 production and promoted ability of DCs to activate allogenic naive CD4^+^ T cells. This effect on DC maturation was mainly dependent on cell-cell contact although NK produced IFN-*γ* and TNF also made contribution [26]. The studies on effect of DC on NK cells by Van Elssen et al. demonstrated that DCs triggered by membrane fraction of* Klebsiella pneumoniae* have the ability to induce CCR7 expression on CD56^dim^CD16^+^  NK cells which promoted their migration in response to lymph node associated chemokine, CCL19. Interestingly, bacterial fragment–matured DC also promoted NK cell activation resulting in higher production of IFN-*γ* and Th1 polarisation [52]. Similarly, chemokines (CXCL10) released from DCs infected with* Mycobacterium tuberculosis* (Mtb) enhanced the migration of NK cells to the site of infection where they can directly destroy Mtb-infected cells or produce IFN-*γ* to stimulate macrophage activation [53]. Moreover, acute systemic infections with* L. monocytogenes *and* Yersinia pestis *identified the regulatory role of NK cells in NK-DC crosstalk. It is found that NK cells are the major source of IL-10, which suppresses IL-12 secretion of DC, thereby preventing further NK cell activation and tissue damage [54].

**Figure 1:**
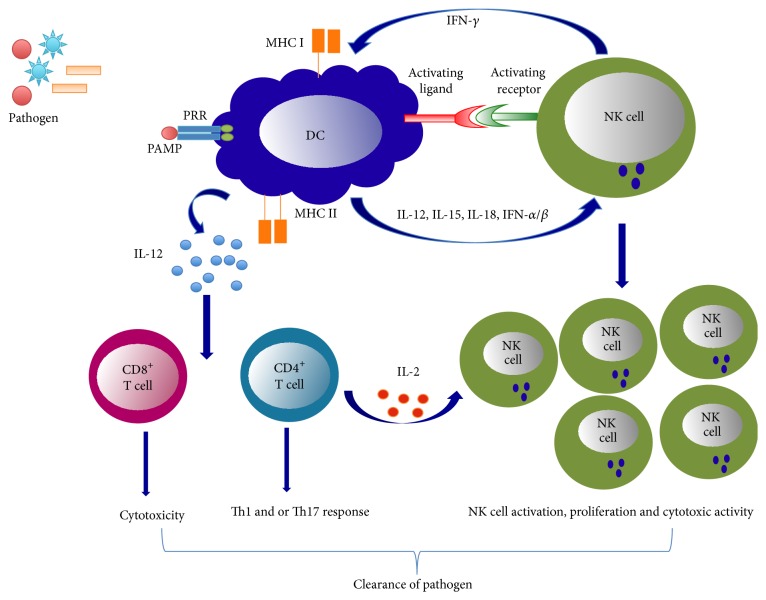
Bidirectional activation of NK cells and DCs for optimal immune response to microbial infection. DCs recognize pathogen-associated molecular patterns (PAMPs) found on the surface of pathogen through the expression of pattern recognition receptor (PRR). Following recognition of pathogen, DCs release cytokines such as IL-12, IL-15, IL-18, and IFN-*α*/*β*. These cytokines produced by DCs as well as the interactions of DC with NK cell activation receptors promote NK cell activation, proliferation, and cytotoxicity. Reciprocally, NK cells release IFN-*γ* to promote DC maturation and release of IL-12. IL-12 produced by DC further promotes CD8^+^ T cell and/or CD4^+^ T cell activation depending upon the nature of pathogen. IL-2 produced by CD4^+^ T cell can also stimulate NK cell activation for optimal immune response.

Our laboratory has studied the modulating effect of NK cells on the function of DCs using an in vivo model of chlamydial lung infection [55, 56].* Chlamydia* is an obligate intracellular bacterium causing various human diseases. The most important protective mechanism in host defense against chlamydial infection is Th1 mediated immunity [57, 58]. Chlamydial infection induces strong NK and NKT cell responses [59–62]. We examined NK and DCs collected from the spleen and lung of* Chlamydiae*-infected mice and analyzed DC phenotype and function by flow cytometry, primary cell culture, and adoptive transfer experiments. We found that depletion of NK cells altered the phenotypic and functional maturation of DCs and promoted infection and pathological reaction in the lung. In line, adoptive transfer of DCs from* Chlamydiae*-infected NK-depleted mice (NK-DC) in contrast to DC from the infected mice without NK depletion (NK+DC), failed to induce type 1 protective immunity in recipient mice after challenge infection. Moreover, NK cells from* Chlamydiae*-infected mice showed enhancing effect on IL-12 production by DC. This effect was depending on NKG2D receptor signaling and IFN *γ* production by NK cells [55]. Furthermore, cytokine analysis of the local tissues of the recipient mice receiving NK-DC compared with those receiving NK+DC exhibited lower levels of Th1 (IFN-*γ*) and Th17 (IL-17) but higher levels of Th2 (IL-4) cytokines. Consistently, NK-DCs were less efficient in directing* Chlamydiae*-specific Th1 and Th17 responses than NK+DCs when co-cultured with CD4^+^ T cells [56]. These studies taken together demonstrate that NK cells modulate DC function to elicit Th1/Th17 immunity during intracellular bacterial infection (Figure 1).

## 6. Conclusion

The generation of pathogen specific adaptive immune response requires efficient interaction between NK cells and DC in the periphery or secondary lymphoid organs. This crosstalk is evident in several models of viral, parasite, and bacterial infection and provides a strong rationale for the combined use of NK cells and DCs in the immunotherapy of chronic infections. The development of therapeutic interventions aimed at enhancing the immune response against infections will require a complete understanding of the molecular mechanisms involved in NK-DC crosstalk and how it becomes disrupted during chronic infection.
